# Clinical outcomes in patients with atrial fibrillation and frailty: insights from the ENGAGE AF-TIMI 48 trial

**DOI:** 10.1186/s12916-020-01870-w

**Published:** 2020-12-24

**Authors:** Chris Wilkinson, Jianhua Wu, Samuel D. Searle, Oliver Todd, Marlous Hall, Vijay Kunadian, Andrew Clegg, Kenneth Rockwood, Chris P. Gale

**Affiliations:** 1 Population Health Sciences Institute, Faculty of Medical Sciences, Newcastle University, Newcastle upon Tyne, NE2 4HH UK; 2 Leeds Institute of Cardiovascular and Metabolic Medicine, University of Leeds, Leeds, UK; 3 Leeds Institute for Data Analytics, University of Leeds, Leeds, UK; 4 MRC Unit for Lifelong Health and Ageing, University College London, London, UK; 5 Geriatric Medicine, Dalhousie University, Halifax, Nova Scotia Canada; 6 Academic Unit for Ageing and Stroke Research, University of Leeds, Leeds, UK; 7 Bradford Teaching Hospitals NHS Foundation Trust, Bradford, UK; 8 Translational and Clinical Research Institute, Faculty of Medical Sciences, Newcastle University, Newcastle upon Tyne, UK; 9 Cardiothoracic Centre, Freeman Hospital, Newcastle upon Tyne Hospitals NHS Foundation Trust, Newcastle Upon Tyne, UK; 10 Department of Cardiology, Leeds Teaching Hospitals NHS Trust, Leeds, UK

**Keywords:** Atrial fibrillation, Frailty, Clinical trial, Anticoagulation, Stroke

## Abstract

**Background:**

Atrial fibrillation (AF) is common in older people with frailty and is associated with an increased risk of stroke and systemic embolism. Whilst oral anticoagulation is associated with a reduction in this risk, there is a lack of data on the safety and efficacy of direct oral anticoagulants (DOACs) in people with frailty. This study aims to report clinical outcomes of patients with AF in the Effective Anticoagulation with Factor Xa Next Generation in Atrial Fibrillation–Thrombolysis in Myocardial Infarction 48 (ENGAGE AF-TIMI 48) trial by frailty status.

**Methods:**

Post hoc analysis of 20,867 participants in the ENGAGE AF-TIMI 48 trial, representing 98.8% of those randomised. This double-blinded double-dummy trial compared two once-daily regimens of edoxaban (a DOAC) with warfarin. Participants were categorised as fit, living with pre-frailty, mild-moderate, or severe frailty according to a standardised index, based upon the cumulative deficit model. The primary efficacy endpoint was stroke or systemic embolism and the safety endpoint was major bleeding.

**Results:**

A fifth (19.6%) of the study population had frailty (fit: *n* = 4459, pre-frailty: *n* = 12,326, mild-moderate frailty: *n* = 3722, severe frailty: *n* = 360). On average over the follow-up period, the risk of stroke or systemic embolism increased by 37% (adjusted HR 1.37, 95% CI 1.19–1.58) and major bleeding by 42% (adjusted HR 1.42, 1.27–1.59) for each 0.1 increase in the frailty index (four additional health deficits). Edoxaban was associated with similar efficacy to warfarin in every frailty category, and a lower risk of bleeding than warfarin in all but those living with severe frailty.

**Conclusions:**

Edoxaban was similarly efficacious to warfarin across the frailty spectrum and was associated with lower rates of bleeding except in those with severe frailty. Overall, with increasing frailty, there was an increase in stroke and bleeding risk. There is a need for high-quality, frailty-specific population randomised control trials to guide therapy in this vulnerable population.

**Trial registration:**

ClinicalTrials.gov NCT00781391. First registered on 28 October 2008

**Supplementary Information:**

The online version contains supplementary material available at 10.1186/s12916-020-01870-w.

## Background

Atrial fibrillation (AF) affects at least 10 million people in Europe [1]. The incidence and prevalence of AF increases with age [2] and is more common in patients with frailty [3, 4], a condition characterised by a decline in a person’s biological reserves and deterioration in physiological mechanisms that render them vulnerable to a range of adverse outcomes [5–9]. Frailty provides an insight into biological age and is more useful than chronological age in predicting adverse events and guiding clinical care [10–14]. Frailty is commonly identified using either a frailty index or a frailty phenotype. The frailty index expresses the proportion of health deficits that a person has accumulated divided by all deficits measured, whereas the phenotype defines frailty as poor performance in three of five criteria (weight loss, exhaustion, weakness, slowness, lack of activity) [7]. There is overlap between the two approaches [15].

Whilst AF is associated with an increased risk of stroke and mortality, an appropriate prescription of oral anticoagulation can reduce the risk of stroke by 64% [16, 17]. Therefore, anticoagulation is recommended for men with AF and a CHA_2_DS_2_-VASc score of 2 or greater and in women with a score of 3 or greater [18, 19]. Large randomised controlled trials have established the efficacy and safety of direct oral anticoagulant medications (DOACs) in comparison to warfarin for stroke prevention in non-valvular AF [20–23], including in people aged over 75 years [24], and they are associated with a per patient cost saving [25]. However, we lack data on the efficacy and safety of DOAC in older people with AF who are also frail [3].

Our three objectives for this study were to estimate the prevalence of frailty in people with AF; describe the association between AF, frailty, and clinical outcomes; and compare the efficacy and safety of edoxaban (a DOAC) to warfarin by frailty category.

## Methods

We constructed a frailty index using data from the Effective Anticoagulation with Factor Xa Next Generation in Atrial Fibrillation–Thrombolysis in Myocardial Infarction 48 (ENGAGE AF-TIMI 48) trial [26]. The use of a frailty index is an established and validated technique for quantifying frailty using the cumulative deficit model and results in substantially better prediction of mortality and other adverse events than age alone [11, 27, 28].

### Study cohort

**Fig. 1:**
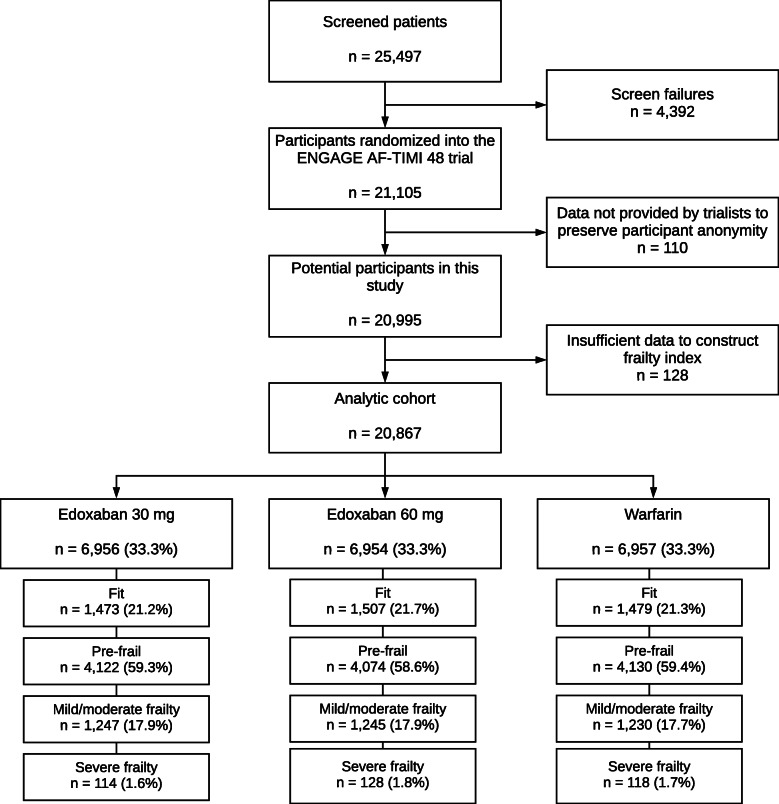
Consort diagram

The design and baseline characteristics of the ENGAGE AF-TIMI 48 study are described elsewhere (NCT00781391) [21, 26]. In brief, this was a randomised, double-blinded, double-dummy trial, in which two once-daily regimens of edoxaban were compared with warfarin in 21,105 patients with AF and a moderate or high risk of stroke. The trial was conducted at 1393 centres across 46 countries. Patients were enrolled from 19 November 2008 to 22 November 2010, and the median follow-up duration was 2.8 years [21]. The protocol and amendments were approved by ethics committees at each participating centre, and all participants provided written informed consent. The dataset supporting the conclusions of this article is available (subject to approval) via application at https://vivli.org. This *post hoc* analysis was approved by an independent review panel. Data were de-identified at source by the trial team, and patients that were deemed by the study team to be at high risk of identification (for example due to a rare medical history) were excluded from the supplied data set. This left 98.8% (*n* = 20,867) of the randomised participants for this analysis (Fig. 1).

### Participants

Patients with AF were eligible for inclusion in the ENGAGE AF-TIMI 48 study if they had documented AF of any duration within the 12 months preceding randomisation and had a CHADS_2_ score of 2 or higher. In this score, 1 point is allocated for each of congestive heart failure, hypertension, diabetes, and an age of 75 years or older. A prior stroke or transient ischaemic attack (TIA) is assigned 2 points. The possible range is 0 to 6, with higher scores associated with an increased stroke risk. Exclusion criteria included AF due to a reversible disorder, an estimated creatinine clearance of less than 30 ml/min, a high risk of bleeding, use of dual antiplatelet therapy, moderate-to-severe mitral stenosis, other indications for anticoagulation therapy; acute coronary syndromes, coronary revascularization, or stroke within 30 days before randomisation; and an inability to adhere to study procedures [21, 26].

### Interventions

The study drugs were warfarin (dose-adjusted to achieve an international normalised ratio [INR] of 2 to 3), edoxaban 30 mg daily, or edoxaban 60 mg daily. Patients were randomised in a 1:1:1 ratio. The allocated dose of edoxaban was halved if the patient had or developed a creatinine clearance of 30–50 ml/min, a body weight of 60 kg or less, or the concomitant use of verapamil or quinidine (or dronedarone, after a protocol amendment on 22 December 2010). Standard dosing was resumed if there was no other indication for dose reduction and the verapamil, quinidine, or dronedarone was stopped.

### Outcomes

The primary efficacy endpoint was the time to the first adjudicated stroke (ischaemic or haemorrhagic) or systemic embolic event. The primary safety endpoint was adjudicated major bleeding during treatment, as defined by the International Society on Thrombosis and Haemostasis [29]. The composite net clinical endpoints were stroke, systemic embolic event, major bleeding, or death; disabling stroke, life-threatening bleeding, or death; and stroke, systemic embolic event, life-threatening bleeding, or death. Given the established association between frailty and mortality [5], we report death as a separate outcome. For composite endpoints involving deaths, individuals were right censored at death in the analysis. An independent clinical endpoint committee, who were blinded to study assignment, adjudicated all deaths and suspected cerebrovascular events, systemic embolic events, myocardial infarctions, bleeding events, and hepatic events [21]. The definitions used by the clinical endpoint committee are provided in the original study protocol [26].

### Blinding

To maintain blinding, each patient received two sets of study drugs—with a placebo matching warfarin for patients in the edoxaban arms or edoxaban for the patients in the warfarin arms. The INR was measured at least monthly, with sham results generated in the edoxaban groups.

### Frailty

We defined frailty using the cumulative deficit model, which identifies deficits in health (such as symptoms, signs, diseases, disabilities, or abnormalities in clinical investigations) on the basis that the more deficits a person has, the more likely that the person is frail [30]. The cumulative deficit model enables calculation of a frailty index as an equally weighted proportion of the number of deficits present in an individual to the total possible. For this study, we constructed a frailty index using the available trial data, calculated at the time of study entry. In line with the established guidance for constructing a frailty index, candidate health deficit variables for inclusion were identified on the basis that they were associated with health status, their prevalence generally increases with age, they do not reach saturation too early (over 80% prevalence before the age of 80), and they cover a range of body systems [31]. Data needed to be available for at least 70% of items for inclusion [32]. The 40 items included in the construction of the frailty index are detailed in Additional file 1: Table S1. The presence or absence of each variable was ascertained from data collected by the trial investigators as part of their protocol-specified assessment, which took place within 30 days of study randomisation [33]. Participants were categorised based upon the frailty index into fit (0 to < 0.12), living with pre-frailty (≥ 0.12 to < 0.24), mild-moderate (≥ 0.24 to < 0.36), and severe frailty (≥ 0.36 to 1.0), based upon thresholds that are commonly used in the literature [11, 34].

### Statistical methods

Baseline characteristics are reported for the frailty groups and by treatment allocation. Continuous variables are presented as medians with interquartile range, and categorical variables as counts and proportions. Event rates for each of the primary and secondary (composite) outcomes were calculated as the number of events per 100 person-years and reported by frailty category and treatment arm.

Cox proportional hazard models were used to (1) test whether each regimen of edoxaban was non-inferior to warfarin for each primary and secondary (composite) outcomes, stratified by frailty category, and (2) quantify the association between frailty category and the primary and secondary (composite) outcomes, with the treatment arm included as an interaction term. The proportional hazards assumption was assessed using Schoenfeld residuals tests. Hazard ratios with 95% confidence intervals (CIs) were reported for each. As the trial participants were randomised by treatment allocation, but not frailty, comparisons by frailty were adjusted for age, sex, race, and region. These limited adjustments were made to preserve the association between frailty and outcomes. Finally, the impact of an increase of 0.1 in frailty index on each clinical outcome for the complete analytical cohort was calculated. Hazard ratios were adjusted for the treatment group and reported alongside estimates further adjusted for age, sex, race, and region, in the whole cohort. A sensitivity analysis was completed for the association between frailty and the clinical outcomes, where frailty index items were removed if they were related to bleeding (history of non-intracranial bleeding and peptic ulcer). Data were analysed in Stata 15.1 and R version 3.4.1.

## Results

### Participants

We include 20,867 participants in the analysis (38.1% women [*n* = 7940]; 86.8% 60 years or older [*n* = 18,119]; 25.5% with paroxysmal AF [*n* = 5311]; Fig. 1, Table 1). Overall, 21.4% (*n* = 4459) of participants were categorised as fit; 59.1% (*n* = 12,326) were pre-frail; 17.8% (*n* = 3722) had mild-moderate frailty; and 1.7% (*n* = 360) had severe frailty (Additional file 1: Fig. S1). The predicted stroke risk was higher with increased frailty (mean CHADS_2_ score: fit 2.39, pre-frail 2.80, mild-moderate frailty 3.37, severe 4.03).

**Table 1:** Baseline characteristics of participants by frailty category

	Frailty category				
	All	Fit	Pre-fail	Mild-moderate	Severe
***n*** **(%)**	20,867	4459 (21.4)	12,326 (59.1)	3722 (17.8)	360 (1.7)
**Mean frailty index (SD)**	0.18 (0.07)	0.09 (0.02)	0.18 (0.03)	0.28 (0.03)	0.40 (0.03)
**Demographics**					
Age, *n* (%)					
< 60 years	2748 (13.2)	739 (16.6)	1671 (13.6)	319 (8.6)	19 (5.3)
60–69 years	5875 (28.2)	1245 (27.9)	3583 (29.1)	979 (26.3)	68 (18.9)
70–79	8716 (41.8)	1795 (40.3)	5111 (41.5)	1641 (44.1)	169 (46.9)
80+ years	3528 (16.9)	680 (15.3)	1961 (15.9)	783 (21.0)	104 (28.9)
Female sex, *n* (%)	7940 (38.1)	1470 (33.0)	4685 (38.0)	1619 (43.5)	166 (46.1)
Region, *n* (%)					
North America	4654 (22.3)	721 (16.2)	2639 (21.4)	1153 (31.0)	141 (39.2)
Latin America	2647 (12.7)	898 (20.1)	1484 (12.0)	252 (6.8)	13 (3.6)
Western Europe	3091 (14.8)	743 (16.7)	1804 (14.6)	492 (13.2)	52 (14.4)
Eastern Europe	7105 (34.0)	1143 (25.6)	4392 (35.6)	1442 (38.7)	128 (35.6)
Asia-Pacific and South Africa	3370 (16.1)	954 (21.4)	2007 (16.3)	383 (10.3)	26 (7.2)
**Clinical**					
Paroxysmal AF, *n* (%)	5311 (25.5)	1073 (24.1)	3195 (25.9)	958 (25.8)	85 (23.6)
Qualifying risk factor, *n* (%)					
Age ≥ 75	8356 (40.0)	1799 (40.3)	4693 (38.1)	1669 (44.8)	195 (54.2)
Prior stroke or TIA	5909 (28.3)	988 (22.2)	3345 (27.1)	1398 (37.6)	178 (49.4)
Congestive heart failure	11,967 (57.3)	1993 (44.7)	7075 (57.4)	2601 (69.9)	298 (82.8)
Diabetes mellitus	7546 (36.2)	825 (18.5)	4478 (36.3)	1989 (53.4)	254 (70.6)
Hypertension	19,454 (93.2)	4083 (91.6)	11,518 (93.4)	3506 (94.2)	347 (96.4)
CHADS_2_ score					
Mean score (SD)	2.83 (0.98)	2.39 (0.67)	2.80 (0.91)	3.37 (1.11)	4.03 (1.19)
≤ 3, *n* (%)	16,167 (77.5)	4099 (91.9)	9739 (79.0)	2193 (58.9)	136 (37.8)
4–6, *n* (%)	4699 (22.5)	360 (8.1)	2587 (21.0)	1528 (41.1)	224 (62.2)
Dose reduction*, *n* (%)	5302 (25.4)	1020 (22.9)	2885 (23.4)	1237 (33.2)	160 (44.4)
Cr clearance ≤ 50 ml/min	3975 (19.2)	613 (13.9)	2119 (17.3)	1083 (29.5)	160 (45.5)
Weight ≤ 60 kg	2063 (9.9)	524 (11.8)	1182 (9.6)	341 (9.2)	16 (4.4)
Use of verapamil or qunidine	701 (3.4)	183 (4.1)	394 (3.2)	116 (3.1)	8 (2.2)
Previous VKA for ≥ 60 days, *n* (%)	12,305 (59.0)	2509 (56.3)	7241 (58.7)	2303 (61.9)	252 (70.0)
**Medication at time of randomisation,** ***n*** **(%)**					
Aspirin	6121 (29.3)	1107 (24.8)	3650 (29.6)	1234 (33.2)	130 (36.1)
Thienopyridine	480 (2.3)	62 (1.4)	264 (2.1)	140 (3.8)	14 (3.9)
Amiodarone	2441 (11.7)	501 (11.2)	1397 (11.3)	489 (13.1)	54 (15.0)
Digoxin or digitalis preparation	6271 (30.1)	1269 (28.5)	3713 (30.1)	1172 (31.5)	117 (32.5)
**Treatment allocation,** ***n*** **(%)**					
Warfarin	6957 (33.3)	1479 (33.2)	4130 (33.5)	1230 (33.0)	118 (32.8)
Edoxaban 30 mg	6956 (33.3)	1473 (33.0)	4122 (33.4)	1247 (33.5)	114 (31.7)
Edoxaban 60 mg	6954 (33.3)	1507 (33.8)	4074 (33.1)	1245 (33.4)	128 (35.6)

*Abbreviations*: *AF* atrial fibrillation, *Cr* creatinine, *SD* standard deviation, *TIA* transient ischaemic attack, *VKA* vitamin K antagonist

*At randomisation

There was a similar number of participants in each treatment arm (warfarin 33.3% [*n* = 6957]; edoxaban 30 mg 33.3% [*n* = 6956]; edoxaban 60 mg 33.3% [*n* = 6954]), and the distribution of frailty category was comparable between treatment arms (Fig. 1). The characteristics of patients in each treatment group, stratified by frailty, are reported in Additional file 1: Table S2.

### Primary outcomes

Across the three treatment arms, 997 patients experienced stroke or systemic embolism (rate per 100 person-years: warfarin 1.73 [95% CI 1.54–1.92]; edoxaban 30 mg 1.95 [1.76–2.15]; edoxaban 60 mg 1.47 [1.30–1.64]; Table 2). There was no difference in stroke or systemic embolism between the treatment arms (Additional file 1: Table S3), including when stratified by frailty category (Table 3). Across the treatment arms, in comparison to the fit group, the average adjusted risk of stroke or systemic embolism over the follow-up period was 84% higher in the group living with mild-moderate frailty and more than double in those living with severe frailty (Table 4). On average over the follow-up period, for each increase of 0.1 in the frailty index (four additional health deficits), the risk of stroke or systemic embolism increased by 37% (adjusted HR 1.37, 1.19–1.58).

**Table 2:** Numbers and rates of outcome events

		Frailty category				
		All	Fit	Pre-frail	Mild-moderate	Severe
**Primary end points**						
Time to the first adjudicated stroke or systemic embolism	Warfarin	333	55	185	83	10
		1.73 (1.54 - 1.92)	1.29 (0.95 - 1.63)	1.61 (1.38 - 1.84)	2.58 (2.03 - 3.14)	3.40 (1.29 - 5.51)
	Edoxaban 30mg	378	56	219	100	3
		1.95 (1.76 - 2.15)	1.34 (0.99 - 1.69)	1.90 (1.64 - 2.15)	3.00 (2.41 - 3.59)	1.04 (0.00 - 2.21)
	Edoxaban 60mg	286	58	151	71	6
		1.47 (1.30 - 1.64)	1.35 (1.00 - 1.70)	1.31 (1.11 - 1.52)	2.13 (1.64 - 2.63)	1.85 (0.37 - 3.34)
Time to the first adjudicated major bleeding during treatment	Warfarin	522	84	298	122	18
		2.76 (2.52 - 3.00)	2.01 (1.58 - 2.44)	2.64 (2.34 - 2.95)	3.85 (3.17 - 4.53)	6.39 (3.44 - 9.35)
	Edoxaban 30mg	249	35	141	60	13
		1.28 (1.12 - 1.44)	0.83 (0.56 - 1.11)	1.22 (1.02 - 1.42)	1.80 (1.34 - 2.25)	4.74 (2.16 - 7.31)
	Edoxaban 60mg	414	82	227	93	12
		2.17 (1.96 - 2.38)	1.94 (1.52 - 2.36)	2.01 (1.75 - 2.27)	2.86 (2.28 - 3.44)	3.80 (1.65 - 5.95)
**Composite net clinical endpoints**						
Stroke, systemic embolic event, major bleeding or death	Warfarin	1462	205	811	393	53
		7.90 (7.50 - 8.31)	4.97 (4.29 - 5.65)	7.34 (6.84 - 7.85)	12.86 (11.59 - 14.14)	19.41 (14.19 - 24.64)
	Edoxaban 30mg	1247	185	686	335	41
		6.58 (6.22 - 6.95)	4.48 (3.83 - 5.13)	6.06 (5.61 - 6.52)	10.35 ( 9.24 - 11.46)	15.71 (10.90 - 20.52)
	Edoxaban 60mg	1321	231	685	361	44
		7.06 (6.68 - 7.44)	5.56 (4.84 - 6.27)	6.16 (5.70 - 6.62)	11.51 (10.32 - 12.70)	14.49 (10.21 - 18.77)
Disabling stroke, life-threatening bleeding, or death	Warfarin	981	124	529	285	43
		5.07 (4.75 - 5.39)	2.91 (2.39 - 3.42)	4.58 (4.19 - 4.98)	8.79 (7.77 - 9.82)	14.58 (10.22 - 18.93)
	Edoxaban 30mg	836	124	448	236	28
		4.26 (3.97 - 4.55)	2.93 (2.42 - 3.45)	3.83 (3.47 - 4.18)	6.95 (6.06 - 7.83)	9.69 ( 6.10 - 13.28)
	Edoxaban 60mg	882	139	454	259	30
		4.52 (4.22 - 4.81)	3.21 (2.68 - 3.74)	3.94 (3.58 - 4.30)	7.76 (6.81 - 8.70)	9.08 ( 5.83 - 12.33)
Stroke, systemic embolic event, life-threatening bleeding, or death	Warfarin	1109	145	603	315	46
		5.80 (5.45 - 6.14)	3.43 (2.87 - 3.98)	5.28 (4.86 - 5.70)	9.88 ( 8.79 - 10.98)	15.91 (11.31 - 20.51)
	Edoxaban 30mg	999	148	542	280	29
		5.16 (4.84 - 5.48)	3.53 (2.96 - 4.10)	4.69 (4.30 - 5.09)	8.42 (7.43 - 9.40)	10.09 ( 6.42 - 13.77)
	Edoxaban 60mg	990	168	501	287	34
		5.12 (4.80 - 5.43)	3.92 (3.33 - 4.52)	4.38 (3.99 - 4.76)	8.70 (7.70 - 9.71)	10.51 ( 6.98 - 14.04)
Death	Warfarin	837	100	447	252	38
		4.27 (3.98 – 4.56)	2.32 (1.86 – 2.77)	3.82 (3.47 – 4.18)	7.62 (6.68 – 8.56)	12.54 (8.56 – 16.53)
	Edoxaban 30mg	736	107	387	217	25
		3.79 (3.52 – 4.06)	2.51 (2.03 – 2.99)	3.37 (3.04 – 3.71)	6.34 (5.50 – 7.18)	8.46 (5.14 – 11.77)
	Edoxaban 60mg	772	121	391	231	29
		3.92 (3.64 – 4.19)	2.77 (2.28 – 3.27)	3.38 (3.05 – 3.72)	6.81 (5.93 – 7.68)	8.71 (5.54 – 11.89)

Each cell shows number, and incidence rates per 100 person years (95% confidence interval)

**Table 3:** The association between oral anticoagulation and outcomes, stratified by frailty category

Unadjusted hazard ratio (95% confidence interval) compared to warfarin (ref), within each frailty category							
Fit		Pre-frail		Mild-moderate frailty		Severe frailty	
Edoxaban 30 mg	Edoxaban 60 mg	Edoxaban 30 mg	Edoxaban 60 mg	Edoxaban 30 mg	Edoxaban 60 mg	Edoxaban 30 mg	Edoxaban 60 mg
*Primary endpoints*							
Time to first adjudicated stroke or systemic embolism							
1.04 (0.71–1.50)	1.03 (0.71–1.49)	1.18 (0.97–1.43)	0.82 (0.66–1.01)	1.17 (0.87–1.56)	0.84 (0.61–1.15)	0.30 (0.08–1.11)	0.54 (0.20–1.50)
Time to adjudicated major bleeding during treatment							
0.42 (0.28–0.62)	0.96 (0.71–1.30)	0.46 (0.38–0.56)	0.76 (0.64–0.90)	0.47 (0.35–0.64)	0.75 (0.57–0.98)	0.74 (0.36–1.52)	0.60 (0.29–1.26)
*Composite net clinical endpoints*							
1. Stroke, systemic embolic event, major bleeding, or death							
0.90 (0.74–1.10)	1.11 (0.92–1.34)	0.83 (0.75–0.92)	0.84 (0.76–0.93)	0.81 (0.70–0.93)	0.90 (0.78–1.03)	0.82 (0.54–1.23)	0.75 (0.50–1.12)
2. Disabling stroke, life-threatening bleeding, or death							
1.01 (0.79–1.30)	1.11 (0.87–1.41)	0.83 (0.74–0.95)	0.86 (0.76–0.97)	0.79 (0.66–0.94)	0.88 (0.74–1.04)	0.66 (0.41–1.07)	0.62 (0.39–0.99)
3. Stroke, systemic embolic event, life-threatening bleeding, or death							
1.03 (0.82–1.30)	1.14 (0.91–1.42)	0.89 (0.79–1.00)	0.83 (0.74–0.93)	0.85 (0.72–1.00)	0.88 (0.75–1.04)	0.64 (0.40–1.02)	0.66 (0.42–1.03)
*Death*							
1.08 (0.83–1.42)	1.20 (0.92–1.56)	0.89 (0.78–1.02)	0.88 (0.77–1.01)	0.83 (0.69–1.00)	0.89 (0.75–1.07)	0.67 (0.41–1.12)	0.69 (0.43–1.12)

**Table 4:** The association between frailty category and clinical outcomes

	Hazard ratio (95% CI)			
	Fit	Pre-frail	Mild-moderate frailty	Severe frailty
*Primary endpoints*				
Time to first adjudicated stroke or systemic embolism				
Unadjusted	1	1.22 (1.03–1.45)	1.93 (1.59–2.35)	1.58 (0.99–2.55)
Adjusted	1	1.22 (0.90–1.65)	1.84 (1.31–2.59)	2.30 (1.17–4.52)
Time to adjudicated major bleeding during treatment				
Unadjusted	1	1.22 (1.04–1.43)	1.75 (1.46–2.09)	3.02 (2.17–4.20)
Adjusted	1	1.32 (1.04–1.68)	1.79 (1.36–2.37)	2.86 (1.72–4.76)
*Composite net clinical endpoints*				
Stroke, systemic embolic event, major bleeding, or death				
Unadjusted	1	1.30 (1.19–1.43)	2.31 (2.09–2.55)	3.29 (2.74–3.96)
Adjusted	1	1.49 (1.28–1.74)	2.45 (2.07–2.90)	3.56 (2.63–4.81)
Disabling stroke, life-threatening bleeding, or death				
Unadjusted	1	1.37 (1.22–1.53)	2.60 (2.30–2.94)	3.69 (2.96–4.59)
Adjusted	1	1.60 (1.32–1.95)	2.88 (2.33–3.55)	4.59 (3.24–6.50)
Stroke, systemic embolic event, life-threatening bleeding, or death				
Unadjusted	1	1.32 (1.19–1.47)	2.49 (2.22–2.78)	3.36 (2.73–4.14)
Adjusted	1	1.56 (1.30–1.87)	2.73 (2.24–3.33)	4.24 (3.04–5.91)
*Death*				
Unadjusted	1	1.40 (1.24–1.58)	2.75 (2.41–3.13)	3.94 (3.13–4.97)
Adjusted	1	1.68 (1.36–2.09)	3.13 (2.48–3.95)	4.97 (3.42–7.23)

Adjustments made for sex, age, race, and region. Interaction by treatment group: not significant

Overall, 1185 participants experienced major bleeding during treatment (rate per 100 person-years: warfarin 2.76, 95% CI 2.52–3.00; edoxaban 30 mg 1.28, 1.12–1.44; edoxaban 60 mg 2.17, 1.96–2.38; Table 2). On average over the follow-up period, bleeding events were 53% lower in patients taking edoxaban 30 mg compared to warfarin (HR 0.47, 95% CI 0.40–0.54) and 21% lower in those taking edoxaban 60 mg (HR 0.79, 0.69–0.89, Additional file 1: Table S3). When stratified by frailty category, edoxaban 30 mg was associated with a reduction in major bleeding compared with warfarin in all but those with severe frailty, and edoxaban 60 mg with a reduction in major bleeding in the pre-frail and mild-moderate frailty groups only (Table 3). Across the treatment arms, the adjusted risk of major bleeding increased with the frailty category, such that for each increase of 0.1 in the frailty index (four additional health deficits), the risk of major bleeding increased by 42% on average over the follow-up period (adjusted HR 1.42, 1.27–1.59).

### Composite net clinical endpoints

Overall, 4030 participants experienced stroke, systemic embolic event, major bleeding, or death (rate per 100 person-years: warfarin 7.90, 95% CI 7.50–8.31; edoxaban 30 mg 6.58, 6.22–6.95; edoxaban 60 mg 7.06, 6.68–7.44; Table 2). Disabling stroke, life-threatening bleeding, or death affected 2699 participants (rates: warfarin 5.07, 4.75–5.39; edoxaban 30 mg 4.26, 3.97–4.55; edoxaban 60 mg 4.52, 4.22–4.81; Table 2). Stroke, systemic embolic event, life-threatening bleeding, or death affected 3098 participants (rates: warfarin 5.80, 5.45–6.14; edoxaban 30 mg 5.16, 4.84–5.48; edoxaban 60 mg 5.12, 4.80–5.43; Table 2).

Compared with warfarin, there was a significant reduction for each of the three composite outcomes associated with the use of edoxaban at both 30-mg and 60-mg dosages (Additional file 1: Table S3). When stratified by frailty category, there was no difference in each of the three composite outcomes according to the treatment arm for those in the fit category (Table 3). In those living with pre-frailty, a reduction in all three composite outcomes was associated with edoxaban 60 mg compared with warfarin. For edoxaban 30 mg, the risk was reduced in composite outcome (1) stroke, systemic embolic event, major bleeding, or death and (2) disabling stroke, life-threatening bleeding, or death—but not for the composite outcome (3) stroke, systemic embolic event, life-threatening bleeding, or death. For those living with mild-moderate frailty, there was a reduction in (1) and (2) with the 30-mg dose, and no difference in the composite outcomes between the use of edoxaban 60 mg and warfarin. Finally, in those with severe frailty, there was a reduction in (2) with edoxaban 60 mg compared to warfarin, and no difference in the composite outcomes between edoxaban 30 mg and warfarin, and for the other composite outcomes for the 60-mg dosage (Table 2).

When modelling frailty index on a continuous scale, there was a significantly increased risk of all three composite outcomes with increasing frailty across all three treatment arms. For each increase of 0.1 in the frailty index (four additional health deficits), the adjusted risks on average over the follow-up period of (1) stroke, systemic embolic event, major bleeding, or death increased by 59% (HR 1.59, 1.48–1.69); (2) disabling stroke, life-threatening bleeding, or death by 72% (1.72, 1.59–1.87); and (3) stroke, systemic embolic event, life-threatening bleeding, or death by 67% (1.67, 1.55–1.80). The overall findings were robust to a sensitivity analysis in which the frailty index was modified to remove factors specifically associated with bleeding risk (Additional file 1: Table S4).

### Mortality

There was a stepwise association between frailty category and mortality, whereby patients with severe frailty had a hazard ratio for mortality of 4.97 (3.42–7.23) compared to the fit group (Table 4). Mortality accounted for a greater proportion of the composite endpoints with increasing frailty category (Table 2).

## Discussion

In this analysis of a large international clinical trial, we have shown that edoxaban is non-inferior to warfarin across the frailty spectrum in stroke prevention. Bleeding events were reduced in patients who received edoxaban except in those living with severe frailty—where standardised bleeding event rates were not statistically significantly different from warfarin. We found that just one in five trial participants had frailty and that frailty was associated with worse clinical outcomes, regardless of treatment arm allocation.

The key finding of the ENGAGE AF-TIMI 48 trial was that edoxaban was associated with lower rates of bleeding and death from cardiovascular causes compared with warfarin, with similar efficacy in stroke and systemic embolism prophylaxis. In our stratified analyses, however, these findings were not upheld for every frailty category. Instead, it appeared that the effect was driven by the pre-frail group, which was the most prevalent frailty category in the trial population. This may relate to a lack of statistical power, particularly as the trend in every subgroup is consistent with the overall trial finding. Even so, we cannot conclude with certainty from this analysis that the overall trial findings are applicable to patients living with severe frailty. Here, those patients were under-represented, even though they are a group at high risk of stroke, and in whom AF is common [3, 4].

The distribution of the frailty scores in this trial is striking. One in five participants was frail, and just one in 50 had severe frailty. This is in contrast with the primary care population of older people with AF, in which over half live with moderate or severe frailty [4]. It is known that health problems tend to accumulate with age and therefore frailty is generally progressive [35], with an average rate of deficit accumulation in community-dwelling older people of 3% per year [36]. With population ageing, the burden of frailty is likely to grow substantially [37], amplifying the need for robust trial data that is specific to people with frailty who are at particular risk of treatment-related harm. The perception of a gap between the representation of people with frailty in trials and the clinical population may explain, at least in part, the relatively low ‘real-world’ prescription rates of oral anticoagulation for eligible patients [38–40] and may reflect clinicians’ fear of causing iatrogenic harm, particularly in people with frailty [3]. This must be considered alongside our finding that death is more common in patients with AF and also frailty—which is well known in a general population [10–14].

We have demonstrated that frailty is associated with worse clinical outcomes regardless of treatment arm allocation. The risk of every trial endpoint was at least doubled for patients with severe frailty compared to the fit group, with the appearance of a ‘dose-response relationship’ despite therapy. This is a population with a high baseline risk of cardiovascular events and death, and a high residual risk remains, despite therapy. This risk is likely to be multifactorial, including non-embolic stroke and death from non-cardiovascular causes that may not be modifiable in the context of advancing multi-organ disease. That the risk of major bleeding on treatment was substantially higher with increasing frailty category may represent a target for improvement. Modifiable bleeding risk factors should be optimised—including a review of concomitant antiplatelet and non-steroidal anti-inflammatory medications [19], as well as a renewed focus on DOAC dosing, which may be complex and is commonly incorrect [41].

A recent randomised controlled trial found that in older Japanese patients, a daily 15-mg dose of edoxaban was superior to placebo in preventing stroke or systemic embolism, without a significantly higher incidence of major bleeding than placebo [42]. These findings are important, but we know that there is a graded degree of risk amongst older people of the same age [4, 7, 10, 30], and there remains a notable lack of generalisable data concerning the outcomes associated with anticoagulation for patients with more advanced frailty [3]. There are many reasons why older people are historically under-represented in clinical trials, including the presence of co-morbidities, communication issues, and physical immobility that limit opportunities for participation [43]. Yet, in view of a high baseline risk of both stroke and iatrogenic harm, there is a strong argument for frailty-specific population randomised trials in this area. In lieu of specific randomised evidence to quantify efficacy and safety in older people with severe frailty, future work modelling outcomes using a combination of epidemiological and trial data may yield interesting insights [44, 45].

This study has strengths, which include the ENGAGE AF-TIMI 48 dataset, in which there were few missing data, a large sample size, and a long follow-up duration [21, 26]. To our knowledge, this study is the first to report the outcomes of such a trial by frailty [3]. As the recruitment of patients with frailty into clinical trials is challenging, these analyses are necessary and important in order to guide clinicians and individualise therapy [46]. Frailty was identified using a conceptually robust and reproducible measure [31], that is increasingly used in understanding the relationship between age and outcomes in clinical trials [10], and allows the risk of adverse outcomes to be defined more precisely than a phenotypic approach [15]. We adjusted the associations between frailty and outcomes for potential confounders. However, we recognise the limitations of our work. In particular, the trial exclusion criteria mean that patients with more severe frailty were excluded, for example patients with a life expectancy of less than 12 months or who were unable to attend for trial visits [26]. This means that the results are not generalisable to the whole population of older people with frailty. Secondly, whilst we did not have access to the complete dataset, the rates of the outcomes were similar to those in the original trial [26]. Thirdly, as the trial was not designed for the analyses that we have undertaken, the analyses stratified by frailty category are likely to be underpowered. Fourthly, data were not available to evaluate phenotypically defined frailty in this dataset. Finally, the study was conducted in the era before specific reversal agents, which may impact upon bleeding severity and associated mortality in future clinical practice.

## Conclusion

Patients with AF taking anticoagulation with warfarin or edoxaban are at substantially higher risk of stroke or systemic embolism, major bleeding during treatment, and death if they also have frailty. We showed important differences in the overall risk of adverse outcomes increasing with frailty, and efficacy was similar between warfarin and edoxaban. Whilst a reduction in bleeding was associated with edoxaban overall, this was not substantiated across all frailty categories, and people with more advanced frailty made up a small proportion of the overall trial population. This highlights the need for high-quality, frailty-specific population randomised controlled trials to guide therapy in this vulnerable population.

## Supplementary Information

**Additional file 1: Figure S1** The distribution of frailty index scores within the analytical cohort. **Table S1** Items included in the frailty index. **Table S2A** Baseline characteristics of participants in the warfarin arm, by frailty category. **Table S2B** Baseline characteristics of participants in the edoxaban 30 mg arm, by frailty category. **Table S2C** Baseline characteristics of participants in the edoxaban 60 mg arm, by frailty category. **Table S3** The association between treatment arm and clinical outcomes. **Table S4** Sensitivity analysis: The association between frailty category and clinical outcomes, with non-intracranial bleeding and peptic ulcer disease excluded from the frailty index.

## Data Availability

The dataset supporting the conclusions of this article is available (subject to approval) via application at https://vivli.org.

## Abbreviations

AF: Atrial fibrillation
CHADS_2_ score: One point for the history of congestive heart failure, hypertension, age ≥ 75 years, and diabetes; two points for stroke or TIA
CHA_2_DS_2_-VASc score: One point for the history of congestive heart failure, hypertension, diabetes, vascular disease history (prior MI, peripheral artery disease, or aortic plaque), age 65–74 years, and female sex; two points for age ≥ 75, history of stroke, or TIA
CI: Confidence interval
Cr: Creatinine
DOAC: Direct oral anticoagulant
ENGAGE AF-TIMI 48: Effective Anticoagulation with Factor Xa Next Generation in Atrial Fibrillation–Thrombolysis in Myocardial Infarction 48
INR: International normalised ratio
SD: Standard deviation
TIA: Transient ischaemic attack
VKA: Vitamin K antagonist
