# Supplementary material for: Clinical outcomes in patients with atrial fibrillation and frailty: insights from the ENGAGE AF-TIMI 48 trial
Source: BMC Med. 2020 Dec 24;18:401. doi: 10.1186/s12916-020-01870-w (PMC7758931; doi:10.1186/s12916-020-01870-w)
Supplement: Supplementary file 1 — Additional file 1: Figure S1 The distribution of frailty index scores within the analytical cohort. Table S1 Items included in the frailty index. Table S2A Baseline characteristics of participants in the warfarin arm, by frailty category. Table S2B Baseline characteristics of participants in the edoxaban 30 mg arm, by frailty category. Table S2C Baseline characteristics of participants in the edoxaban 60 mg arm, by frailty category. Table S3 The association between treatment arm and clinical outcomes. Table S4 Sensitivity analysis: The association between frailty category and clinical outcomes, with non-intracranial bleeding and peptic ulcer disease excluded from the frailty index. [file 12916_2020_1870_MOESM1_ESM.docx]

Supplementary material: additional file 1

**Outcomes for people with atrial fibrillation and frailty: insights from the**

**ENGAGE AF-TIMI 48 trial**

**Figure S1. The distribution of frailty index scores within the analytical cohort**


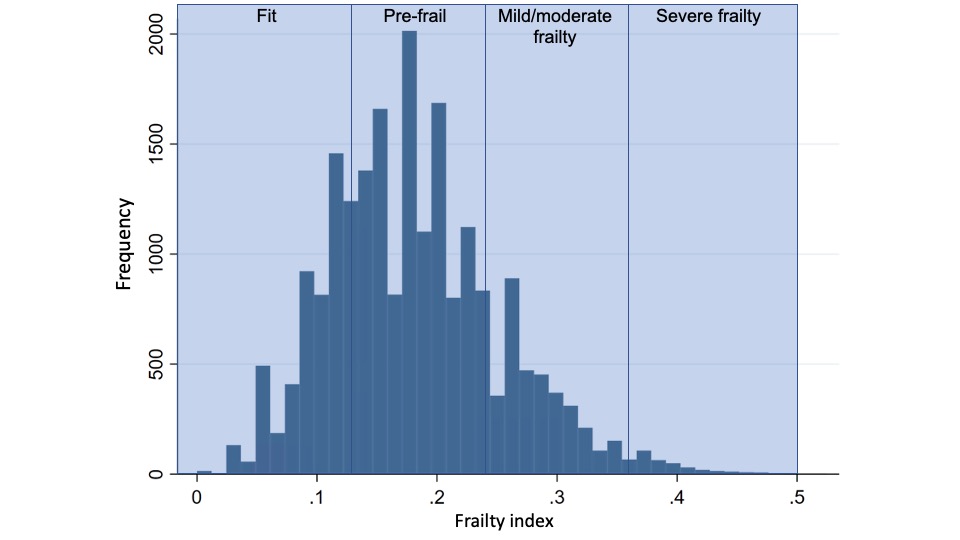


**Table S1. Items included in the frailty index**

| **Variable** | **Prevalence** | | **Cut-points** |  |
| --- | --- | --- | --- | --- |
|  | **%** | **n** |  |  |
| **Co-morbidities, history of:** |  |  |  |  |
| Non-intracranial bleeding | 6.0 | 1,254 | None 0, Any 1 |  |
| Peptic ulcer disease | 6.2 | 1,291 | None 0, Any 1 |  |
| Renal disease^1^ | 11.6 | 2,444 | None 0, Any 1 |  |
| Dyslipidaemia | 52.4 | 10,999 | None 0, Any 1 |  |
| Diabetes requiring treatment | 36.1 | 7,583 | None 0, Any 1 |  |
| Fracture | 14.6 | 3,074 | None 0, Any 1 |  |
| Increased risk of falls | 4.3 | 894 | None 0, Any 1 |  |
| Hyper or hypothyroidism | 11.9 | 2,495 | None 0, Any 1 |  |
| Neuropsychiatric disease^2^ | 7.8 | 1,636 | None 0, Any 1 |  |
| Osteoporosis | 6.3 | 1,330 | None 0, Any 1 |  |
| Rheumatoid arthritis | 2.1 | 434 | None 0, Any 1 |  |
| Ischaemic or embolic stroke | 18.7 | 3,928 | None 0, Any 1 |  |
| Transient ischaemic attack | 11.7 | 2,452 | None 0, Any 1 |  |
| Congestive heart failure | 57.4 | 12,050 | None 0, Any 1 |  |
| Coronary artery disease | 33.6 | 7,054 | None 0, Any 1 |  |
| Carotid disease | 6.4 | 1,345 | None 0, Any 1 |  |
| Peripheral arterial disease | 4.0 | 842 | None 0, Any 1 |  |
| Valvular heart disease^3^ | 21.0 | 4,400 | None 0, Any 1 |  |
| Left ventricular hypertrophy (on 12-lead electrocardiogram) | 16.3 | 3,429 | None 0, Any 1 |  |
|  |  |  |  |  |
| **Laboratory values**^4^ |  |  |  |  |
| Urinary bacteria | 17.0 | 3,578 | Normal 0, Abnormal 1 |  |
| Urinary yeast | 1.4 | 294 | Normal 0, Abnormal 1 |  |
| Serum Bilirubin | 5.9 | 1,208 | Normal 0, Abnormal 1 |  |
| Serum creatinine^1^ | 18.6 | 3,833 | Normal 0, Abnormal 1 |  |
| Serum glucose | 20.4 | 4,203 | Normal 0, Abnormal 1 |  |
| Serum urea | 20.4 | 2,895 | Normal 0, Abnormal 1 |  |
| Serum alkaline phosphatase | 11.8 | 2,382 | Normal 0, Abnormal 1 |  |
| Haemoglobin | 9.7 | 1,985 | Normal 0, Abnormal 1 |  |
| White blood cells | 4.2 | 855 | Normal 0, Abnormal 1 |  |
| Lymphocytes | 10.0 | 2,020 | Normal 0, Abnormal 1 |  |
| Neutrophils | 5.6 | 1,127 | Normal 0, Abnormal 1 |  |
| Platelets | 10.1 | 2,015 | Normal 0, Abnormal 1 |  |
| Occult haematuria | 13.1 | 2,648 | None 0, Any 1 (trace+) |  |
| Pulse pressure | 80.3 | 16,816 | 0: 30-40. 1: <30 \| >40 |  |
|  |  |  |  |  |
| **EuroQoL questionnaire** |  |  |  |  |
| Mobility | 0.0 57.7  0.5 42.1  1.0 0.3 | 6,258  4,566  32 | 0: no problems  0.5: moderate problems  1: confined to bed |  |
| Self-care | 0.0 87.1  0.5 12.3  1.0 0.6 | 9,451  1,331  68 | 0: no problems  0.5: moderate problems  1: unable |  |
| Usual activities | 0.0 67.2  0.5 30.8  1.0 2.0 | 7,289  3,343  212 | 0: no problem  0.5: moderate problems  1: Unable to perform |  |
| Pain or discomfort | 0.0 51.3  0.5 46.5  1.0 2.2 | 5,554  5,043  238 | 0: no problem  0.5: moderate  1: severe |  |
| EQ-5D | 0.0 46.0  1.0 54.0 | 4,757  5,578 | 0: >median (75)  1: <median |  |
|  |  |  |  |  |
| **Physical examination** |  |  |  |  |
| Systolic blood pressure | 0.0 43.2  0.5 26.4  1.0 30.5 | 9,044  5,524  6,388 | 0: 90-120mmHg  0.5: 120-140mmHg  1: <90 \| >140mmHg |  |
| Heart rate | 0.0 85.0  1.0 15.1 | 17,810  3,156 | 0: 60-100bpm  1: <60 \| >100bpm |  |
| Notes:  1: An eGFR <30ml/min was an exclusion criterion for the ENGAGE AF-TIMI 48 trial  2: Dementia, depression, Parkinson's disease, schizophrenia, seizures  3: Mechanical valve replacement, or moderate/severe mitral stenosis were exclusion criteria  4: For laboratory values, those outside the normal laboratory reference range were scored one, using the range reported for the individual within the dataset  **Abbreviations** bpm: beats per minute; mmHg: millimetres of mercury | | | | |

**Table S2A. Baseline characteristics of participants in the warfarin arm, by frailty category**

|  | **All** | **Fit** | **Pre-frail** | **Mild-moderate** | **Severe** |
| --- | --- | --- | --- | --- | --- |
| **n (%)** | 6957 | 1479 (21.3) | 4130 (59.4) | 1230 (17.7) | 118 (1.7) |
| **Mean FI (SD)** | 0.18 (0.07) | 0.09 (0.02) | 0.18 (0.03) | 0.28 (0.03) | 0.40 (0.03) |
|  |  |  |  |  |  |
| **Demographics** |  |  |  |  |  |
| Age, n (%) |  |  |  |  |  |
| < 60 | 941 (13.5) | 243 (16.4) | 580 (14.0) | 110 (8.9) | 8 (6.8) |
| 60 - 69 | 1965 (28.2) | 426 (28.8) | 1188 (28.8) | 327 (26.6) | 24 (20.3) |
| 70 - 79 | 2869 (41.2) | 581 (39.3) | 1727 (41.8) | 509 (41.4) | 52 (44.1) |
| 80+ | 1182 (17.0) | 229 (15.5) | 635 (15.4) | 284 (23.1) | 34 (28.8) |
| Female sex, n (%) | 2608 (37.5) | 455 (30.8) | 1596 (38.6) | 502 (40.8) | 55 (46.6) |
| Region, n (%) |  |  |  |  |  |
| North America | 1553 (22.3) | 247 (16.7) | 865 (20.9) | 391 (31.8) | 50 (42.4) |
| Latin America | 885 (12.7) | 301 (20.4) | 484 (11.7) | 97 (7.9) | 3 (2.5) |
| Western Europe | 1017 (14.6) | 254 (17.2) | 579 (14.0) | 169 (13.7) | 15 (12.7) |
| Eastern Europe | 2369 (34.1) | 367 (24.8) | 1495 (36.2) | 464 (37.7) | 43 (36.4) |
| Asia-Pacific & South Africa | 1133 (16.3) | 310 (21.0) | 707 (17.1) | 109 (8.9) | 7 (5.9) |
|  |  |  |  |  |  |
| **Clinical** |  |  |  |  |  |
| Paroxysmal AF, n (%) | 1760 (25.3) | 353 (23.9) | 1053 (25.5) | 326 (26.5) | 28 (23.7) |
| Qualifying risk factor, n (%) |  |  |  |  |  |
| Age ≥75 | 2777 (39.9) | 606 (41.0) | 1548 (37.5) | 561 (45.6) | 62 (52.5) |
| Prior stroke or TIA | 1981 (28.5) | 328 (22.2) | 1137 (27.5) | 453 (36.8) | 63 (53.4) |
| Congestive heart failure | 4004 (57.6) | 671 (45.4) | 2391 (57.9) | 849 (69.0) | 93 (78.8) |
| Diabetes melitus | 2490 (35.8) | 272 (18.4) | 1465 (35.5) | 667 (54.2) | 86 (72.9) |
| Hypertension | 6489 (93.3) | 1341 (90.7) | 3870 (93.7) | 1165 (94.7) | 113 (95.8) |
| CHADS2 score mean (SD) | 2.83 (0.98) | 2.40 (0.69) | 2.80 (0.92) | 3.37 (1.11) | 4.07 (1.27) |
| ≤3, n (%) | 5382 (77.4) | 1349 (91.2) | 3264 (79.0) | 729 (59.3) | 40 (33.9) |
| 4 - 6, n (%) | 1574 (22.6) | 130 (8.8) | 866 (21.0) | 500 (40.7) | 78 (66.1) |
| Dose reduction* n (%) | 1769 (25.4) | 339 (22.9) | 961 (23.3) | 420 (34.1) | 49 (41.5) |
| Cr clearance ≤ 50 ml/min | 1327 (19.2) | 207 (14.1) | 708 (17.3) | 364 (30.0) | 48 (41.7) |
| Weight ≤ 60 kg | 698 (10.0) | 164 (11.1) | 415 (10.1) | 113 (9.2) | 6 (5.1) |
| Use of verapamil or qunidine | 219 (3.1) | 61 (4.1) | 121 (2.9) | 36 (2.9) | 1 (0.8) |
| Previous VKA for ≥60 days n (%) | 4084 (58.7) | 835 (56.5) | 2432 (58.9) | 735 (59.8) | 82 (69.5) |
| Medication* n (%) |  |  |  |  |  |
| Aspirin | 2075 (29.8) | 373 (25.2) | 1211 (29.3) | 440 (35.8) | 51 (43.2) |
| Thienopyridine | 161 (2.3) | 19 (1.3) | 77 (1.9) | 58 (4.7) | 7 (5.9) |
| Amiodarone | 810 (11.6) | 170 (11.5) | 469 (11.4) | 160 (13.0) | 11 (9.3) |
| Digoxin or digitalis preparation | 2153 (30.9) | 421 (28.5) | 1296 (31.4) | 398 (32.4) | 38 (32.2) |
| **Abbreviations** AF: atrial fibrillation, Cr: creatinine, SD: standard deviation, TIA: transient ischaemic attack, VKA: vitamin K antagonist. * at randomisation | | | | | |

**Table S2B. Baseline characteristics of participants in the edoxaban 30mg arm, by frailty category**

|  | **All** | **Fit** | **Pre-frail** | **Mild-moderate** | **Severe** |
| --- | --- | --- | --- | --- | --- |
| **n (%)** | 6956 | 1473 (21.2) | 4122 (59.3) | 1247 (17.9) | 114 (1.6) |
| **Mean FI (SD)** | 0.18 (0.07) | 0.09 (0.02) | 0.18 (0.03) | 0.28 (0.03) | 0.40 (0.03) |
|  |  |  |  |  |  |
| **Demographics** |  |  |  |  |  |
| Age, n (%) |  |  |  |  |  |
| < 60 | 872 (12.5) | 248 (16.8) | 531 (12.9) | 91 (7.3) | 2 (1.8) |
| 60 - 69 | 1998 (28.7) | 409 (27.8) | 1226 (29.7) | 344 (27.6) | 19 (16.7) |
| 70 - 79 | 2901 (41.7) | 589 (40.0) | 1692 (41.0) | 565 (45.3) | 55 (48.2) |
| 80+ | 1185 (17.0) | 227 (15.4) | 673 (16.3) | 247 (19.8) | 38 (33.3) |
| Female sex, n (%) | 2695 (38.7) | 530 (36.0) | 1572 (38.1) | 545 (43.7) | 48 (42.1) |
| Region, n (%) |  |  |  |  |  |
| North America | 1547 (22.2) | 219 (14.9) | 901 (21.9) | 378 (30.3) | 49 (43.0) |
| Latin America | 882 (12.7) | 297 (20.2) | 503 (12.2) | 76 (6.1) | 6 (5.3) |
| Western Europe | 1039 (14.9) | 244 (16.6) | 626 (15.2) | 156 (12.5) | 13 (11.4) |
| Eastern Europe | 2368 (34.0) | 382 (25.9) | 1443 (35.0) | 505 (40.5) | 38 (33.3) |
| Asia-Pacific & South Africa | 1120 (16.1) | 331 (22.5) | 649 (15.7) | 132 (10.6) | 8 (7.0) |
|  |  |  |  |  |  |
| **Clinical** |  |  |  |  |  |
| Paroxysmal AF, n (%) | 1814 (26.1) | 360 (24.4) | 1109 (26.9) | 316 (25.4) | 29 (25.4) |
| Qualifying risk factor, n (%) |  |  |  |  |  |
| Age ≥75 | 2776 (39.9) | 587 (39.9) | 1589 (38.5) | 538 (43.1) | 62 (54.4) |
| Prior stroke or TIA | 1977 (28.4) | 324 (22.0) | 1114 (27.0) | 489 (39.2) | 50 (43.9) |
| Congestive heart failure | 3924 (56.4) | 645 (43.8) | 2315 (56.2) | 869 (69.7) | 95 (83.3) |
| Diabetes melitus | 2521 (36.2) | 283 (19.2) | 1495 (36.3) | 667 (53.5) | 76 (66.7) |
| Hypertension | 6482 (93.2) | 1358 (92.2) | 3842 (93.2) | 1172 (94.0) | 110 (96.5) |
| CHADS2 score mean (SD) | 2.83 (0.97) | 2.39 (0.67) | 2.78 (0.91) | 3.39 (1.10) | 3.89 (1.07) |
| ≤3, n (%) | 5417 (77.9) | 1363 (92.5) | 3272 (79.4) | 731 (58.6) | 51 (44.7) |
| 4 - 6, n (%) | 1538 (22.1) | 110 (7.5) | 850 (20.6) | 515 (41.3) | 63 (55.3) |
| Dose reduction* n (%) | 1769 (25.4) | 340 (23.1) | 979 (23.8) | 397 (31.8) | 53 (46.5) |
| Cr clearance ≤ 50 ml/min | 1299 (18.9) | 196 (13.4) | 705 (17.2) | 340 (27.7) | 58 (51.3) |
| Weight ≤ 60 kg | 687 (9.9) | 184 (12.5) | 398 (9.7) | 101 (8.1) | 4 (3.5) |
| Use of verapamil or qunidine | 241 (3.5) | 56 (3.8) | 141 (3.4) | 42 (3.4) | 2 (1.8) |
| Previous VKA use for ≥60 days n (%) | 4123 (59.3) | 846 (57.4) | 2403 (58.3) | 789 (63.3) | 85 (74.6) |
| Medication* n (%) |  |  |  |  |  |
| Aspirin | 1998 (28.7) | 360 (24.4) | 1187 (28.8) | 409 (32.8) | 42 (36.8) |
| Thienopyridine | 148 (2.1) | 16 (1.1) | 87 (2.1) | 41 (3.3) | 4 (3.5) |
| Amiodarone | 783 (11.3) | 160 (10.9) | 467 (11.3) | 139 (11.1) | 17 (14.9) |
| Digoxin or digitalis preparation | 2061 (29.6) | 422 (28.6) | 1215 (29.5) | 388 (31.1) | 36 (31.6) |
| \| **Abbreviations** AF: atrial fibrillation, Cr: creatinine, SD: standard deviation, TIA: transient ischaemic attack, VKA: vitamin K antagonist. * at randomisation \| \| --- \| | | | | | |

**Table S2C. Baseline characteristics of participants in the edoxaban 60mg arm, by frailty category**

| **Frailty category** | **All** | **Fit** | **Pre-frail** | **Mild-moderate** | **Severe** |
| --- | --- | --- | --- | --- | --- |
| **n (%)** | 6954 | 1507 (21.7) | 4074 (58.6) | 1245 (17.9) | 128 (1.8) |
| **Mean FI (SD)** | 0.18 (0.07) | 0.09 (0.02) | 0.18 (0.03) | 0.28 (0.03) | 0.39 (0.03) |
|  |  |  |  |  |  |
| **Demographics** |  |  |  |  |  |
| Age, n (%) |  |  |  |  |  |
| < 60 | 935 (13.4) | 248 (16.5) | 560 (13.7) | 118 (9.5) | 9 (7.0) |
| 60 - 69 | 1912 (27.5) | 410 (27.2) | 1169 (28.7) | 308 (24.7) | 25 (19.5) |
| 70 - 79 | 2946 (42.4) | 625 (41.5) | 1692 (41.5) | 567 (45.5) | 62 (48.4) |
| 80+ | 1161 (16.7) | 224 (14.9) | 653 (16.0) | 252 (20.2) | 32 (25.0) |
| Female sex, n (%) | 2637 (37.9) | 485 (32.2) | 1517 (37.2) | 572 (45.9) | 63 (49.2) |
| Region, n (%) |  |  |  |  |  |
| North America | 1554 (22.3) | 255 (16.9) | 873 (21.4) | 384 (30.8) | 42 (32.8) |
| Latin America | 880 (12.7) | 300 (19.9) | 497 (12.2) | 79 (6.3) | 4 (3.1) |
| Western Europe | 1035 (14.9) | 245 (16.3) | 599 (14.7) | 167 (13.4) | 24 (18.8) |
| Eastern Europe | 2368 (34.1) | 394 (26.1) | 1454 (35.7) | 473 (38.0) | 47 (36.7) |
| Asia-Pacific & South Africa | 1117 (16.1) | 313 (20.8) | 651 (16.0) | 142 (11.4) | 11 (8.6) |
|  |  |  |  |  |  |
| **Clinical** |  |  |  |  |  |
| Paroxysmal AF, n (%) | 1737 (25.0) | 360 (23.9) | 1033 (25.4) | 316 (25.4) | 28 (21.9) |
| Qualifying risk factor, n (%) |  |  |  |  |  |
| Age ≥75 | 2803 (40.3) | 606 (40.2) | 1556 (38.2) | 570 (45.8) | 71 (55.5) |
| Prior stroke or TIA | 1951 (28.1) | 336 (22.3) | 1094 (26.9) | 456 (36.6) | 65 (50.8) |
| Congestive heart failure | 4039 (58.1) | 677 (44.9) | 2369 (58.1) | 883 (70.9) | 110 (85.9) |
| Diabetes melitus | 2535 (36.5) | 270 (17.9) | 1518 (37.3) | 655 (52.6) | 92 (71.9) |
| Hypertension | 6483 (93.2) | 1384 (91.8) | 3806 (93.4) | 1169 (93.9) | 124 (96.9) |
| CHADS2 score mean (SD) | 2.84 (0.97) | 2.39 (0.66) | 2.81 (0.90) | 3.36 (1.12) | 4.12 (1.21) |
| ≤3, n (%) | 5367 (77.2) | 1387 (92.0) | 3203 (78.6) | 732 (58.8) | 45 (35.2) |
| 4 - 6, n (%) | 1586 (22.8) | 120 (8.0) | 871 (21.4) | 512 (41.1) | 83 (64.8) |
| Dose reduction* n (%) | 1764 (25.4) | 341 (22.6) | 945 (23.2) | 420 (33.7) | 58 (45.3) |
| Creatinine clearance ≤ 50 ml/min | 1349 (19.6) | 210 (14.0) | 706 (17.5) | 379 (30.9) | 54 (43.5) |
| Weight ≤ 60 kg | 678 (9.8) | 176 (11.7) | 369 (9.1) | 127 (10.2) | 6 (4.7) |
| Use of verapamil or qunidine | 241 (3.5) | 66 (4.4) | 132 (3.2) | 38 (3.1) | 5 (3.9) |
| Previous VKA use for ≥60 days n (%) | 4098 (58.9) | 828 (54.9) | 2406 (59.1) | 779 (62.6) | 85 (66.4) |
| Medication* n (%) |  |  |  |  |  |
| Aspirin | 2048 (29.5) | 374 (24.8) | 1252 (30.7) | 385 (30.9) | 37 (28.9) |
| Thienopyridine | 171 (2.5) | 27 (1.8) | 100 (2.5) | 41 (3.3) | 3 (2.3) |
| Amiodarone | 848 (12.2) | 171 (11.3) | 461 (11.3) | 190 (15.3) | 26 (20.3) |
| Digoxin or digitalis preparation | 2057 (29.6) | 426 (28.3) | 1202 (29.5) | 386 (31.0) | 43 (33.6) |
| \| \| **Abbreviations** AF: atrial fibrillation, Cr: creatinine, SD: standard deviation, TIA: transient ischaemic attack, VKA: vitamin K antagonist. * at randomisation \| \| --- \| \| \| --- \| --- \| | | | | | |

**Table S3. The association between treatment arm and clinical outcomes**

|  |  | Hazard ratio (95% CI) | | | |
| --- | --- | --- | --- | --- | --- |
|  |  | Warfarin | Edoxaban 30mg | | Edoxaban 60mg |
| *Primary end points* |  |  |  | |  |
| Time to first adjudicated stroke or systemic embolism |  |  |  | |  |
| Unadjusted |  | 1 | 1.13 (0.97-1.31) | | 0.85 (0.73-1.00) |
| Adjusted |  | 1 | 1.17 (0.78-1.76) | | 0.99 (0.64-1.52) |
| Time to adjudicated major bleeding during treatment |  |  |  | |  |
| Unadjusted |  | 1 | 0.47 (0.40-0.54) | | 0.79 (0.69-0.89) |
| Adjusted |  | 1 | 0.38 (0.25-0.57) | | 0.97 (0.68-1.38) |
|  |  |  |  | |  |
| *Composite net clinical endpoints:* |  |  |  | |  |
| Stroke, systemic embolic event, major bleeding or death |  |  |  | |  |
| Unadjusted |  | 1 | 0.83 (0.77-0.90) | | 0.89 (0.83-0.96) |
| Adjusted |  | 1 | 0.85 (0.69-1.04) | | 0.96 (0.78-1.17) |
| Disabling stroke, life-threatening bleeding, or death |  |  |  | |  |
| Unadjusted |  | 1 | 0.84 (0.77-0.92) | | 0.89 (0.81-0.97) |
| Adjusted |  | 1 | 0.99 (0.76-1.28) | | 0.99 (0.77-1.27) |
| Stroke, systemic embolic event, life-threatening bleeding, or death |  |  |  | |  |
| Unadjusted |  | 1 | 0.89 (0.82-0.97) | | 0.88 (0.81-0.96) |
| Adjusted |  | 1 | 1.04 (0.82-1.32) | | 0.97 (0.77-1.23) |
|  |  |  |  | |  |
| *Death* |  |  |  | |  |
| Unadjusted |  | 1 | 0.89 (0.80-0.98) | | 0.92 (0.83-1.01) |
| Adjusted |  | 1 | 1.05 (0.80-1.38) | | 1.02 (0.78-1.34) |
| Adjustments made for sex, age, race and region |  |  | |  | |

**Table S4. Sensitivity analysis: The association between frailty category and clinical outcomes, with non-intracranial bleeding and peptic ulcer disease excluded from the frailty index**

|  |  | Hazard ratio (95% CI), frailty category | | | |
| --- | --- | --- | --- | --- | --- |
|  |  | Robust | Pre-frail | Mild-moderate | Severe |
| *Primary end points* |  |  |  |  |  |
| Time to first adjudicated stroke or systemic embolism |  |  |  |  |  |
| Unadjusted |  | 1 | 1.27 (1.05-1.55) | 1.90 (1.54-2.35) | 1.73 (1.13-2.64) |
| Adjusted |  | 1 | 1.11 (0.80-1.54) | 1.61 (1.13-2.30) | 1.76 (0.91-3.41) |
| Time to adjudicated major bleeding during treatment |  |  |  |  |  |
| Unadjusted |  | 1 | 1.21 (1.02-1.45) | 1.67 (1.37-2.02) | 3.04 (2.24-4.13) |
| Adjusted |  | 1 | 1.33 (1.01-1.74) | 1.77 (1.31-2.38) | 2.69 (1.65-4.37) |
|  |  |  |  |  |  |
| *Composite net clinical endpoints:* |  |  |  |  |  |
| Stroke, systemic embolic event, major bleeding or death |  |  |  |  |  |
| Unadjusted |  | 1 | 1.32 (1.20-1.46) | 2.22 (2.00-2.48) | 3.50 (2.95-4.15) |
| Adjusted |  | 1 | 1.52 (1.28-1.81) | 2.34 (1.94-2.81) | 3.47 (2.60-4.62) |
| Disabling stroke, life-threatening bleeding, or death |  |  |  |  |  |
| Unadjusted |  | 1 | 1.38 (1.22-1.57) | 2.50 (2.18-2.85) | 4.16 (3.40-5.08) |
| Adjusted |  | 1 | 1.76 (1.40-2.21) | 2.86 (2.25-3.63) | 4.99 (3.56-7.00) |
| Stroke, systemic embolic event, life-threatening bleeding, or death |  |  |  |  |  |
| Unadjusted |  | 1 | 1.33 (1.19-1.50) | 2.38 (2.11-2.70) | 3.61 (2.98-4.38) |
| Adjusted |  | 1 | 1.61 (1.31-1.98) | 2.58 (2.07-3.21) | 4.22 (3.06-5.81) |
|  |  |  |  |  |  |
| *Death* |  |  |  |  |  |
| Unadjusted |  | 1 | 1.41 (1.23-1.62) | 2.63 (2.28-3.05) | 4.40 (3.55-5.45) |
| Adjusted |  | 1 | 1.93 (1.49-2.49) | 3.19 (2.44-4.17) | 5.60 (3.88-8.08) |
| Adjustments made for sex, age, race and region | | | | | |
